# Grant Report on Anxiety-CBT: Dimensional Brain Behavior Predictors of CBT Outcomes in Pediatric Anxiety

**DOI:** 10.20900/jpbs.20200005

**Published:** 2020-02-28

**Authors:** Julie E. Premo, Yanni Liu, Emily L. Bilek, K. Luan Phan, Christopher S. Monk, Kate D. Fitzgerald

**Affiliations:** 1 Department of Psychiatry, University of Michigan, Ann Arbor, MI 48109, USA; 2 Department of Psychiatry and Behavioral Health, The Ohio State University, Columbus, OH 43210, USA; 3 Department of Psychology, University of Michigan, Ann Arbor, MI 48109, USA

**Keywords:** anxiety, cognitive behavioral therapy, fMRI, cognitive control, acute threat, emotion processing, Research Domain Criteria

## Abstract

In the following grant report, we describe initial and planned work supported by our National Institute of Mental Health R01-funded, Research Domain Criteria (RDoc) informed project, “Dimensional Brain Behavior Predictors of CBT Outcomes in Pediatric Anxiety”. This project examines response to cognitive behavioral therapy (CBT) in a large sample of anxiety-affected and low-anxious youth ages 7 to 18 years using multiple levels of analysis, including brain imaging, behavioral performance, and clinical measures. The primary goal of the project is to understand how brain-behavioral markers of anxiety-relevant constructs, namely acute threat, cognitive control, and their interaction, associate with CBT response in youth with clinically significant anxiety. A secondary goal is to determine whether child age influences how these markers predict, and/or change, across varying degrees of CBT response. Now in its fourth year, data from this project has informed the examination of (1) baseline (i.e., pre-CBT) anxiety severity as a function of brain-behavioral measures of cognitive control, and (2) clinical characteristics of youth and parents that associate with anxiety severity and/or predict response to CBT. Analysis of brain-behavioral markers before and after CBT will assess mechanisms of CBT effect, and will be conducted once the data collection in the full sample has been completed. This knowledge will help guide the treatment of clinically anxious youth by informing for whom and how does CBT work.

## INTRODUCTION

### Background

Anxiety disorders are among the most prevalent of childhood psychopathologies and tend to emerge early in childhood, and can become chronic, leading to depression, substance abuse, and school-drop out [1–4]. To reduce clinically significant anxiety and prevent its sequelae, patients must be effectively treated early. However, as many as 40% of clinically anxious children who receive the first line intervention, cognitive behavior therapy (CBT), fail to get better, with up to 60% of treated patients continuing to experience impairment from residual symptoms [5]. A larger percentage (80%) of pediatric anxiety patients benefit from CBT combined with medication [5], however, many patients, parents and clinicians prefer an initial trial of CBT only, before considering medication options [6]. While reasonable, this practice can delay effective treatment and increase risk for chronic anxiety and associated disability [7]. With little information to determine whether a given child will benefit from CBT alone, clinical management rests on trial and error, potentially wasting valuable time. To identify the children most likely to benefit from CBT *and* accelerate the development of novel treatments to help those who fail to respond to CBT alone, objective measures are needed to (1) predict CBT outcome in child anxiety patients and (2) quantify mechanistic changes underlying varying degrees of CBT response.

Ideally, these predictors/mechanisms would be defined by objective biological measures—relevant to the pathophysiology of anxiety—to guide clinical management using currently available treatments, and to serve as targets for the development of novel interventions [8]. However, several barriers have prevented translation of clinical trial and neuroscience research to achieve these goals. First, most clinical trial and neuroimaging research has relied on categorical diagnosis, even though high rates of comorbidity and developmental fluidity between types of anxiety (especially in children) suggest common underlying mechanisms [9]. Second, intervention trials typically assess treatment outcomes based on change in symptoms defined by diagnostic status, rather than correction of underlying biological dysfunction or stimulation of compensatory mechanisms. Notably, CBT “targets” abnormalities of cognition and behavior that are shared across categorical anxiety disorders, suggesting that, when effective, CBT alters the same biological systems [10]. Third, while the same systems may be involved in CBT response, most biological systems undergo dramatic developmental change when anxiety first presents: childhood and adolescence. Indeed, recent work shows that biological mechanisms for anxiety may shift with patient age [11]. Yet, development-by-biology interactions on the expression and treatment of pediatric anxiety remain relatively unknown.

To address these gaps in clinical translational research, the National Institute of Mental Health launched the RDoC (Research Domain Criteria) project, grounded on three principles with high relevance for understanding predictors and mechanisms of response to CBT: (1) clinically significant anxiety is mediated by abnormal brain circuitry; (2) multiple levels of analysis (e.g., functional and structural neuroimaging, behavior, etc.) can be used to index brain circuit function; and (3) such data will yield biomarkers that align with clinical signs and symptoms to guide treatment [8]. To identify biologically based predictors and mechanisms of CBT response in pediatric anxiety, these principles must be considered within a developmental framework, since identification of sensitive periods for RDoC constructs may enable the administration of specific interventions when they are most likely to be effective [12,13].

Aligned with RDoC, “Dimensional Brain Behavior Predictors of CBT Outcomes in Pediatric Anxiety” is a five-year study, currently in its fourth year. We measure anxiety severity and brain-behavioral markers of RDoC constructs, acute threat and cognitive control, in 210 anxious youths with clinically significant (i.e., causing moderate to high levels of impairment) “anxiety, worry, and/or nervousness” and 70 low anxious youth. Two thirds of the anxious participants are randomized to receive a 12-week manualized cognitive behavioral intervention, heavily inspired by the Coping Cat and Cat Project protocols [14–17]. The other third enter a 12-week active comparison condition, Relaxation and Mentorship Therapy (RMT) (anxious youth-control) designed to control for non-specific effects of therapy (e.g., weekly visits, therapeutic alliance, adherence to a treatment protocol) [18]. This control group allows us to isolate the specific effects of CBT on brain function and structure. The comparison sample of 70 low anxious youth are enrolled to control for non-specific effects of time (e.g., change over 12 weeks, repetition of MRI scan). To test our developmental hypotheses, we enroll subjects across a wide age range, 7– 18 years old. Data is collected in all subjects at two time points, approximately 12-weeks apart, using a multi-modal assessment with fMRI, DTI, and behavioral performance to index brain-behavioral correlates of anxiety (i.e., acute threat, cognitive control and their interactions), and dimensional, transdiagnostic measures of anxiety. Pre- to post-therapy change in anxiety severity serves as the primary outcome variable in linear regression analyses testing brain-behavioral correlates to (a) predict CBT outcome, (b) characterize brain changes following CBT, and (c) examine age effects on predictors/mechanisms of CBT effect.

### Participant Characteristics

As of February 2020, 168 children (115 female) have been recruited from the community and University of Michigan medical clinics, and enrolled in the study. Subject age ranges from 7 to 18 years (M = 11.73, SD = 3.27). Children are predominantly from middle to upper-middle class families (77.1% reported a family income of at least $75,000), are 75% Caucasian, 4.8% African American, 5.4% Asian, 13.1% multiracial (1.8% prefer not to say), and are 96% non-hispanic. Of the 126 enrolled anxious youth, 28 withdrew from the study, whereas 4 out of 42 enrolled in the low anxious group withdrew.

Clinical characteristics of the youth sample are comprehensively evaluated. In order to study symptoms dimensionally, according to RDoC guidelines, continuous measures of anxiety symptom severity, impairment, and improvement across treatment were assessed by masters-level clinicians using the PARS [19] and Children’s Global Assessment Scale [20]. In-person assessment of clinically anxious and low anxious youth participants also included a semi-structured clinical diagnostic interview, the Kiddie Schedule for Affective Disorders and Schizophrenia for School-Age Children Present and Lifetime Version [21], a widely used, reliable and valid diagnostic screening tool for children ages 6 to 18 years that comprehensively assesses anxiety as well as other disorders relevant to exclusion and inclusion criteria. For inclusion as a clinically anxious participant, a diagnosis with at least one of the following anxiety disorders was required: Separation Anxiety Disorder, Social Anxiety Disorder, Generalized Anxiety Disorder, Panic Disorder (PD), Specific Phobia, or Other Specified Anxiety Disorder. Exclusion criteria for clinically anxious participants include a primary diagnosis of Posttraumatic Stress Disorder (PTSD) or Obsessive Compulsive Disorder (OCD) given that current nosology of DSM-5 do not include PTSD and OCD as “anxiety disorders”, difficulty/inappropriateness in modifying Coping Cat/Cat Project for PTSD and OCD; and that OCD may involve different brain circuit abnormalities than tapped by our AT and CC-AT probes. Children must also have no (1) personal current or past manic/hypomanic episode or psychotic symptoms; (2) autism spectrum disorder at assessment, active suicidal ideation or be inappropriate for the CBT treatment. As part of the screening interview, a detailed account of prior treatment history (type, duration, dose, adherence) is recorded and used to determine clinical appropriateness of CBT treatment. Exclusionary criteria for control youth includes a history of past or current mental illness, based on the K-SADS-PL. Exclusionary criteria for all subjects includes (1) use of any medication, prescription or non-prescription, with psychotropic effects, (2) active alcohol and substance dependence, and (3) cognitive dysfunction (traumatic brain injury, mental retardation [IQ < 80]). In addition, clinical consensus is also required to confirm that exposure-based CBT for anxiety is the most clinically and developmentally appropriate treatment for each patient participant. Youth also provide self-report on measures of anxiety symptoms (Multi-dimensional Anxiety Scale for Children [22]) and depression symptoms (Child Depression Inventory [23]). Parents provide report on youth emotional and behavioral functioning using the Child Behavioral Checklist [24] and the Screen for Child Anxiety Related Disorders [25] and also complete questionnaires pertaining to their own symptomatology and parenting behavior, including anxiety symptoms (Beck Anxiety Inventory [26]), depressive symptoms (Beck Depression Inventory [27]) and accommodation of child anxiety symptoms (Family Accommodation Scale—Anxiety [28]). Finally, youth also complete a behavioral task measuring response inhibition (Go/No-Go task).

### Overall Project Structure: A Multiple-PI, Team Science Approach

The lead site for the project is the University of Michigan (UM), under Principal Investigators (PIs) Dr. Kate Fitzgerald and Dr. Christopher Monk, who works closely with PI Dr. K. Luan Phan at The Ohio State University. Multiple PIs Drs. Fitzgerald, Monk, & Phan have prior collaboration experience, successfully completing a NIH R01 project (“Brain Markers of Anxiety Disorders and SSRI Treatment in Children and Adolescents”), resulting in many peer-reviewed co-authored publications [29–35]. Thus, in the current project, they work together closely, taking advantage of previous professional collaboration and knowledge of each other’s expertise, all while making unique but equal contributions to the research. All recruitment and assessments (clinical, behavioral, neuroimaging) take place at the University of Michigan. Clinical and behavioral assessments occur in the UM Department of Psychiatry, and neuroimaging data is collected at the UM Functional MRI Laboratory.

MPI Dr. Fitzgerald is a child psychiatrist with expertise in the cognitive neuroscience of anxiety and OCD in children and adolescents. She serves as the Phil F. Jenkins Endowed Research Professor of Depression, Associate Professor of Psychiatry, and Co-Director of the Pediatric Anxiety Clinic within the division of Child and Adolescent Psychiatry at the University of Michigan. As PI and co-I on numerous NIH- and foundation-funded studies, Dr. Fitzgerald has an established track record of publication, collaboration, and leadership. Dr. Fitzgerald serves as contact PI for the study. She supervises staff in the collection, quality control, and analysis of both neuroimaging and treatment outcomes data, and oversees IRB administration, subject recruitment, and clinical assessment of subjects. With Drs. Monk and Phan, she is responsible for dissemination of results through conference presentations and manuscripts.

MPI Dr. Monk is a developmental psychologist with expertise in the affective and cognitive neuroscience of developmental psychopathology. He serves as Professor in the Departments of Psychology and Psychiatry, and Research Professor at the Survey Research Center Institute for Social Research, and the Center for Human Growth and Development at the University of Michigan. Dr. Monk assists in the quality control, analysis and interpretation of combined neuroimaging and clinical data, as well as dissemination of results.

MPI Dr. K. Luan Phan is an adult psychiatrist and expert in affective neuroscience of anxiety and neural mechanisms and predictors of clinical interventions. At the beginning of the project, he served as the Center on Depression and Resilience Endowed Professor, the Director of the Mood and Anxiety Disorders Research Program (MADRP) and Associate Head for Clinical and Translational Research in the Department of Psychiatry at the University of Illinois at Chicago. More recently, he has moved to The Ohio State University where he is Professor and Chair of the Department of Psychiatry and Behavioral Health; Chief of Psychiatry Services for the Health System; and the Charles F. Sinsabaugh Chair in Psychiatry. Dr. Phan contributes to data analyses, interpretation and dissemination of results.

Dr. Yanni Liu is a Research Assistant Professor in the Department of Psychiatry at the University of Michigan with extensive training and expertise in cognitive neuroscience and multimodal neuroimaging techniques. Dr. Liu processes and inspects all fMRI data in addition to conducting analyses. She has presented data from the study at national conferences, and is currently preparing a manuscript as lead author to describe the relationship between brain response to errors and youth anxiety across the clinical-nonclinical spectrum of severity.

Dr. Emily Bilek is a Clinical Assistant Professor and licensed clinical psychologist in the Department of Psychiatry at the University of Michigan. She is board certified in Behavioral and Cognitive Psychology and specializes in the treatment of anxiety and OCD in children and adolescents. Dr. Bilek led the writing of the CBT and RMT treatment manuals with Dr. Fitzgerald, with input from our consultant, Dr. John Piacentini, an internationally renowned youth CBT expert. She also supervises CBT delivery across study therapists to ensure fidelity to the manuals, and mentors study team members in preparing research presentations pertaining to clinical outcomes.

### Rationale, Goals, and Aims

#### Rationale

The broadest contribution of the present study will be to characterize the brain circuitry that predicts better or worse CBT response and to understand how CBT alters the brain to reduce anxiety. This contribution is significant for two reasons: (1) it will provide an important first step paving the way for clinicians to eventually use brain-behavioral measures to advise patients on likelihood of response to CBT monotherapy; and (2) by understanding how CBT modifies the brain to reduce anxiety, it will set the stage for the development of biologically-based treatments that improve brain function to reduce symptoms. By studying brain-behavioral indices of cognitive control, acute threat and their interaction before and after CBT delivery, we believe we can establish objective measures to (1) predict CBT outcome in patients presenting with anxiety and (2) quantify mechanistic changes underlying CBT response to accelerate the development of more effective treatments.

#### Goals

The research has two fundamental goals. The first goal is to determine if individual differences in CBT-relevant brain-behavioral functions lead to variation in CBT outcomes. The RDoC constructs of Cognitive Control (CC), Acute Threat (AT) and the Cognitive Control-Acute Threat interaction (CC-AT) shape the selection of putative frontolimbic brain-behavior targets of CBT for the proposed study. CC is widely held to be implemented by reactivity of frontal regions including the dorsal anterior cingulate cortex [dACC], dorsolateral prefrontal cortex [DLPFC] and ventrolateral prefrontal cortex [VLPFC]; AT includes reactivity in regions such as the amygdala and insula; and interactions between CC and AT are captured by connectivity between these regions. Given that CBT facilitates control over acute threat to enable effective regulation of anxiety, we hypothesize that brain-behavioral markers of CC, AT, and CC-AT will predict and characterize mechanisms of CBT effect [33,35]. The second goal is to address if development contributes to this variation in the relationship between brain behavioral markers of these constructs and CBT outcomes. Based on emerging evidence (including our own pilot data) showing later development of neural substrate for CC compared to earlier increase in brain reactivity to threat [36–38], we hypothesize that these markers will differentially relate to CBT effect, depending on patient age. By bridging developmental neuroscience and randomized clinical trial research, this project will clarify the relationship between frontolimbic brain-behavioral functions (CC, AT, and CC-AT interaction), CBT effect and developmental stage. This knowledge will pave the way towards (1) use of brain-behavioral predictors to identify children most likely to be benefit from CBT and (2) customization of next generation, hypothesis-driven therapy for pediatric anxiety (e.g., cognitive training) targeted to marker profiles of different patients, at different ages.

#### Aims

##### Aim 1:

How do dimensional brain-behavior indices of AT predict CBT outcome, change following CBT and interact with development (age, pubertal status) in terms of CBT response?

##### Aim 2:

How do dimensional brain-behavior indices of CC predict CBT outcome, change following CBT, and interact with development (age, pubertal status) in terms of CBT response?

##### Aim 3:

How do dimensional brain-behavior indices of the CC-AT interaction predict CBT outcome, change following CBT and interact with development (age, pubertal status) in terms of CBT response?

## STUDIES LINKING BRAIN-BEHAVIORAL MARKERS OF COGNITIVE CONTROL AND ACUTE THREAT WITH YOUTH ANXIETY

### Background & Methods

Establishing brain-behavioral predictors and mechanisms of CBT effect will require data from the full sample of youth who have completed treatment to achieve adequate statistical power; however, associations between brain-behavioral markers and anxiety severity remain only poorly understood, especially in youth. Thus, we have begun to pursue interim analyses examining baseline brain-behavioral markers of CC, AT and CC-AT across the full spectrum of anxiety severity represented by our sample. Specifically, we can examine brain activation using fMRI during behavioral tasks that assess these constructs, and their relation to the primary dependent variable, anxiety severity. This work will bolster existing findings suggesting these domains are highly relevant to the development and maintenance of anxiety disorders. Moreover, we can begin to examine relations between task-based fMRI data, diffusion-weighted measures of structural connectivity, resting state functional connectivity, and anxiety severity to test models for analyzing multi-level data.

Insufficient prefrontal cortical control over subcortically mediated threat reactivity has long been postulated as a core mechanism for anxiety, but emerging work suggests that abnormalities of CC may occur in anxious youth even in the absence of overtly threatening stimuli [39–41]. Broadly defined, CC enables the flexible adjustment of behavior by constraining attention to task-relevant stimuli following cognitively salient events (e.g., errors, cognitive interference) [42]. CC is served by the cingulo-opercular and fronto-parietal networks for task control, active during a wide range of cognitive processes (e.g., errors, interference between competing cognitive stimuli [cognitive conflict], response inhibition, task switching)[43–45]. Neuroimaging research shows that the cingulo-opercular network activates to salient stimuli, including errors, interference and other inhibitory control demands on cognitive tasks, and signals for engagement of the frontal-parietal network to adjust behavior and thereby improve performance [45–49]. Impoverished recruitment of the fronto-parietal network during cognitive interference, without emotional context, relates to higher levels of anxiety in children and adults [41,50,51].

It is not entirely clear whether impoverished recruitment of cortical networks for CC underlie the expression of anxiety, but it has been suggested that CC deficits could drive anxiety. That is, inability to engage task control networks could associate to difficulty detecting or identifying automatic anxious thoughts (e.g., worry) as excessive, inappropriate “thinking errors” [52] and/or hinder recruitment of CC resources that may enable healthy individuals to move on from anxious thoughts. On the other hand, neurophysiological research comparing youth and adults with anxiety disorders to healthy controls indicating error-related hyperactivity of the dACC node of the cingulo-opercular network may suggest that over-sensitive brain response to errors could provoke or increase anxiety [53].

**Figure 1.**
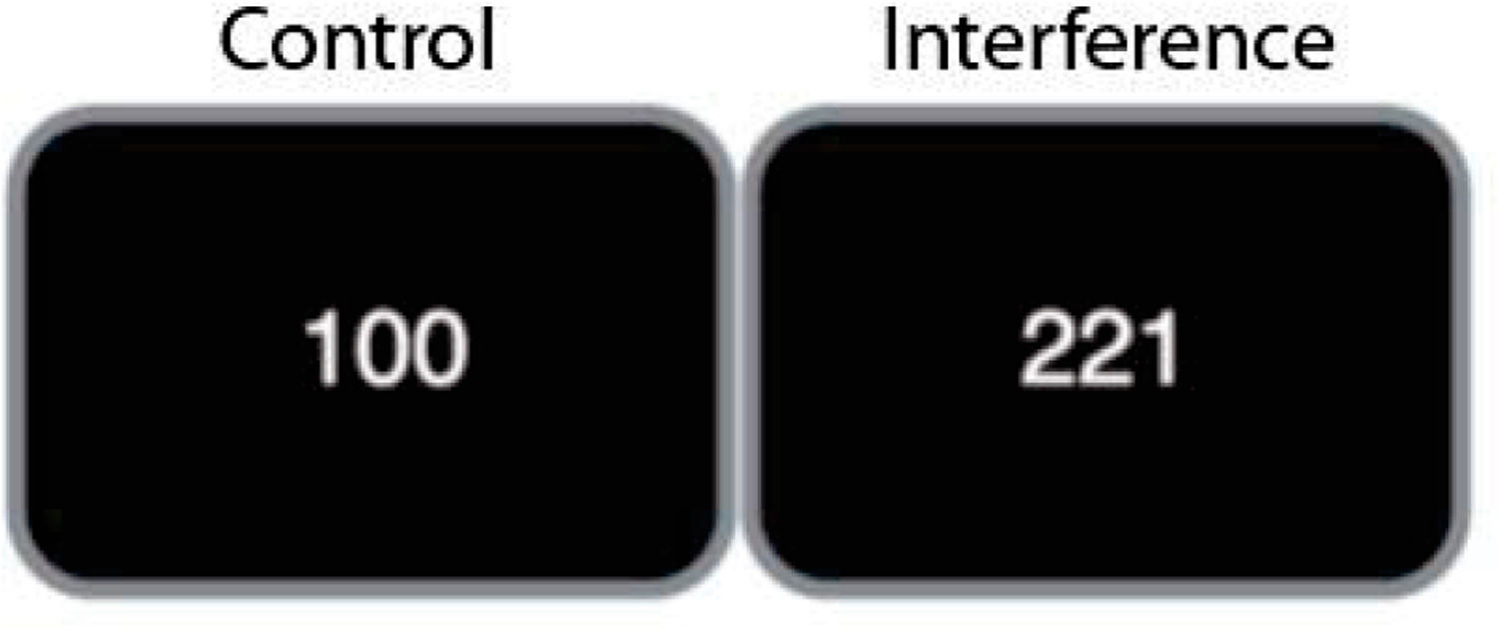
Examples of conditions of the MSIT task.

In our study, components of CC, error and interference processing, are assessed using the Multisource Interference Task (MSIT, Figure 1) [54]. The MSIT probes CC by eliciting interference between task-relevant and prepotent, but task irrelevant response tendencies. High interference trials also elicit errors, making the MSIT paradigm a useful probe of brain response to both cognitive interference and errors. The MSIT was originally developed to activate dACC but also engages VLPFC and DLPFC [44], including in pediatric samples [55,56]. The task requires subjects to identify the unique number among three digits, “1”, “2”, or “3” (e.g., for “311,” the target is “3”) by making a key press with one of three fingers, corresponding to the ordinal value of the target number: “1” → index finger, “2” → middle finger, “3” → ring finger. Interference occurs when the target number position is incongruent with its ordinal value (e.g., “3” presented at the 1st position) and with different, flanking numbers (e.g., “11”). In the control condition, the target number is always presented in a position compatible with its ordinal value (e.g., “1” presented in the first position) and flanked by zeroes (e.g., “100”). Our event-related version of the MSIT allows for separation of fMRI BOLD signal associated with correct incongruent, correct congruent and error trials. We have extensive experience with this task, as demonstrated by our preliminary data, and have shown that it reliably activates the cingulo-opercular and fronto-parietal networks for task control in children and adolescents with and without anxiety symptoms [55,56].

In contrast to CC which has been relatively less studied in the anxiety disorders, especially in youth, a larger body of evidence links anxiety to altered neural circuitry underlying reactivity to acute threat, particularly within limbic regions such as the amygdala and insula. Some of this work also suggests *decreased* prefrontal cortical engagement couples with *increased* limbic region reactivity to threat to lead to anxiety, and an accumulating body of evidence shows altered engagement of pgACC regulatory control of AT through inverse connectivity with limbic centers, such as the amygdala [48,49]. These lines of evidence point towards atypical interactions of neural circuitry of CC-AT in the anxiety disorders, while other work shows that even passive processing of threat (i.e., without regulatory demands), associates with exaggerated amygdala and insula response in clinically anxious individuals from adolescence into adulthood [57–62]. Consistent with the RDoC framework, greater amygdala activation to threat has been found to correlate with anxiety severity in youth across the normal to abnormal range [63].

These converging lines of evidence implicate neural networks for CC, AT and CC-AT in the expression of anxiety, and emerging research suggests that assessment of function in these networks could be used to predict treatment response and/or measure neural mechanisms of treatment effect. Data suggests that amygdala activity in response to threat predicts response to CBT in anxious youth [64] and neuroimaging research suggests that the therapeutic effect of the exposure component of CBT involves desensitization of amygdala reactivity to threat [65]. Given that this reactivity may be greater at younger ages [63], but see [66], and that anxious children may benefit more from exposure than anxious adolescents [67], we posit that developmental stage may be a particularly important context in which to understand neural mechanisms of CBT effect. Specifically, we hypothesize that AT is not only a target of CBT for pediatric anxiety, but that it will be particularly important for CBT benefit in younger relative to older patients. Finally, given evidence of an imbalance between prefrontal cortical substrate for regulatory control and amygdala-based circuits for processing threat in anxiety [59,68,69] it is also possible that effective CBT targets *increases* in prefrontal cortical regions for CC and *decreases* in amygdala reactivity threat to enable patients to more effectively regulate anxious responding. Importantly, age-related increases in inverse connectivity amygdala-pgACC and— vmPFC connectivity have been previously documented [63,66], further underscoring the importance of considering age when examining how CBT may alter prefrontal cortical control of threat reactivity in association with reduction in anxiety symptoms.

**Figure 2.**
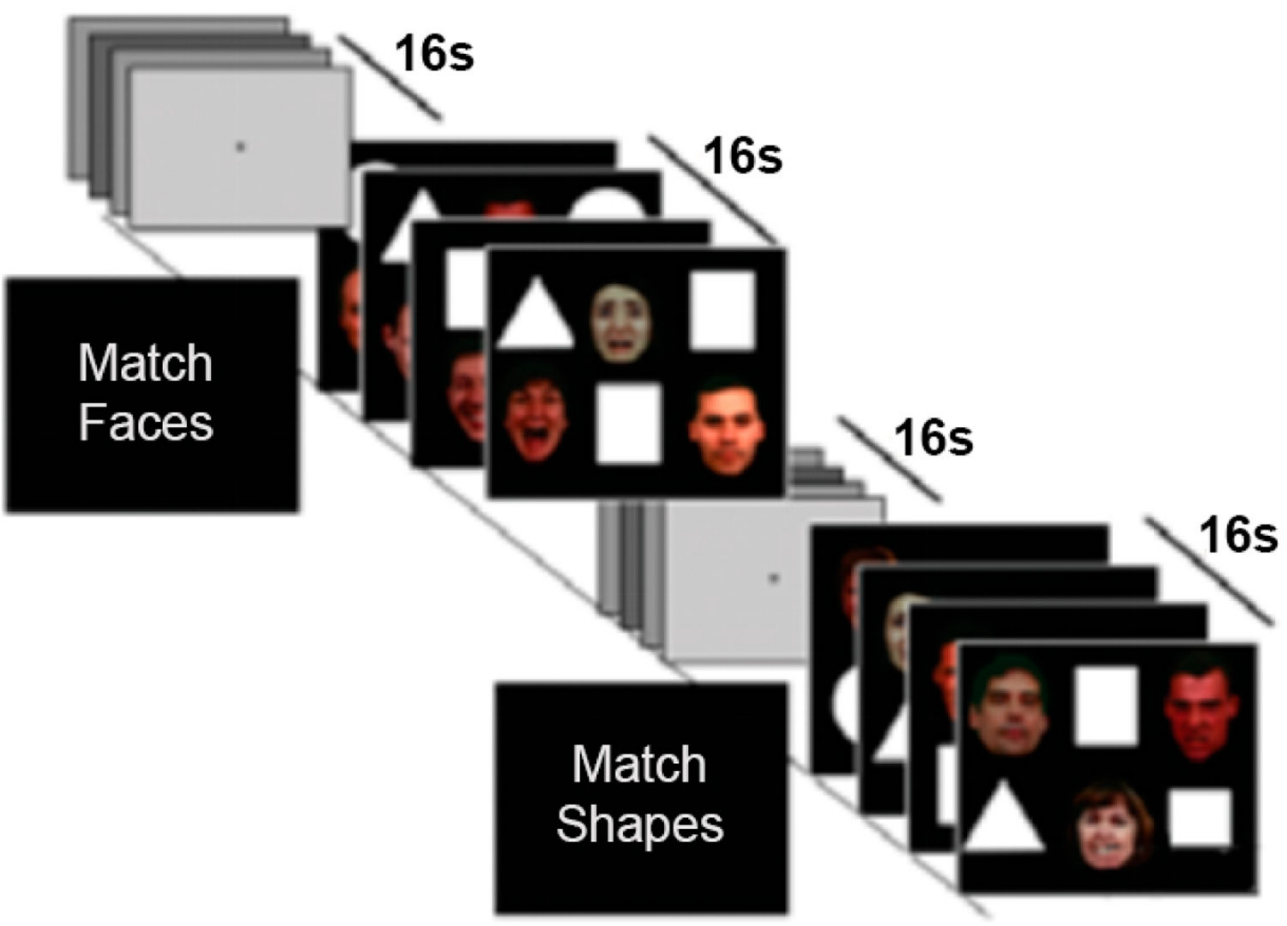
EFSAT task procedure.

To further understand the neural processes linking CBT effect with neural reactivity, we chose the Emotional Faces Shifted Attention Task (EFSAT, Figure 2) [29]. The EFSAT is a validated, effective probe of AT and CC-AT processing, previously reported from our laboratory [29,30]. In a block-related design, participants view two ‘types’ of stimuli on each trial aggregated in blocks according to task instruction (a screen that precedes each block): (1) “Match Faces”—a trio of faces and are instructed to match one of the two faces (bottom) that expressed the same emotion as the target face flanked by a trio of shapes in the same field of view; and (2) “Match Shapes”—same instructions but with a trio of shapes flanked by a trio of faces in the same field of view; block order was pseudorandomized within and across subjects. The target and congruent probe face display one of four expressions (angry, fearful, happy, neutral) and the other (incongruent) probe face always display a neutral (or happy if the target is neutral) expression. Faces were selected from the Gur/UPenn stimulus set [70]. The Match Faces instruction requires subjects to attend to angry and fearful (relative to happy and neutral) facial expressions to probe acute threat, while the Match Shapes instruction requires subjects to ignore emotional faces in order to attend to shapes to assess the interaction between CC and AT processing.

### Progress Report

#### Study 1. Task-control network brain responses to error associate with anxiety across the spectrum of severity

Cognitive control enables the flexible adjustment of behavior by constraining attention to task-relevant stimuli and may be critically impaired in anxiety. Fronto-parietal and cingulo-opercular regions mediate cognitive control, as shown by activation of these regions during a range of cognitive tasks [54,71]. In youth with anxiety disorders, compared to healthy controls, altered response during errors, induced by conflict, suggests impaired ability to engage cognitive control. Unknown is whether capacity to engage task control networks, in response to errors, associates with anxiety across the spectrum of severity. Errors were induced during fMRI scanning using the MSIT in 76 youth (59 females; 12.8 ± 3.2, 7.2–17.9 years; 54 youth with clinical anxiety; 22 youth with subclinical to low anxiety) and tested for association with scores on the clinician-administered Pediatric Anxiety Rating Scale (PARS, 16.4 ± 7.4, 0– 26) [19]. Activation maps for error relative to correct trials were generated in SPM; effect of anxiety severity, measured with the PARS, on brain response to errors was tested in a whole-brain analysis across subjects, covarying age, gender, motion and behavioral performance. In post-hoc analysis, parameter estimates were extracted from brain regions associated with anxiety severity across subjects, and tested for association with anxiety severity among clinically affected youth (i.e., those who met criteria for anxiety disorder(s) on structured clinical interview). Errors activated frontal-parietal and cingulo-opercular task-control regions across subjects. Error-related activation in bilateral parietal lobes and posterior medial frontal cortex associated with higher anxiety symptom severity across subjects.

**Figure 3.**
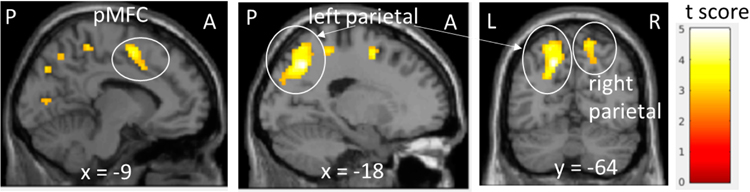
Error-related brain activations associated with anxiety symptom severity across subjects. Data are presented at a peak threshold of *p* < 0.005 uncorrected and cluster-wise threshold of *p* < 0.05 (corrected for false discovery rate). The color bar shows t score. A, anterior; P, posterior; L, left; R, right; pMFC, posterior medial frontal cortex; *x*, *y*, MNI coordinates.

In youth with clinically significant anxiety, more activation in both left and right parietal regions associated with greater anxiety severity (Figure 3). Additionally, behavioral performance on the MSIT indicated increased anxiety severity was associated with a slower reaction time and a larger reaction time congruency effect across subjects, controlling for child age and gender. The association of higher levels of anxiety—across the full spectrum of severity—with greater error-related activation of task-control brain regions links neural substrate for cognitive control with the expression of anxiety along a normal to abnormal continuum. Future research is needed to determine how decreasing or increasing error-related engagement of task control regions (e.g., following CBT) may associated with changes in the expression of anxiety symptoms in youth.

### Papers and Presentations

- Liu Y, Monk CS, Hanna GL, Phan KL, Fitzgerald KD. Dorsal anterior cingulate response to conflict associates with anxiety across the spectrum of severity. Poster presented at the annual meeting of the Society for Biological Psychiatry; 2018 May; New York, NY, USA [72].
- Fitzgerald KD, Liu Y, Morrison C; Hanna GL, Phan KL, Monk CS. Neural networks for cognitive control may underlie response to cognitive behavioral therapy in clinically anxious youth. Oral presentation at: the American College of Neuropsychopharmacology; 2018 Dec; Hollywood, FL, USA [73].
- Liu Y, Morrison C; Hanna GL, Phan KL, Monk CS, Fitzgerald KD, Neural networks for cognitive control may underlie response to cognitive behavioral therapy in clinically anxious youth. Oral presentation at the Anxiety Disorders Association of America, 2019 March; Chicago, IL, USA [74].
- Fitzgerald, KD. Task control networks and cognitive behavioral therapy response in clinically anxious youth. Oral presentation at the European Association of Clinical Psychology and Psychological Treatment; 2019 Oct 31–Nov 2; Dresden, Germany [75].
- Liu, Y, Premo, J, Monk, CS, Phan, KL, Fitzgerald, KD. Task-control network brain responses to error associate with anxiety across the spectrum of severity. To be presented at the annual Anxiety and Depression Association of America Conference, 2020 Mar 19–22; San Antonio, TX, USA [76].
- Liu, Y, Premo, J, Phan, KL, Monk, CS, Fitzgerald, KD. Greater task-control network activation to errors predicts greater anxiety across the non-clinical to clinical range of severity. To be presented at the annual meeting of the Society of Biological Psychiatry; 2020 Apr 30– May 2; New York, NY, USA [77].

### Planned Research

Consistent with the primary aims of this project, future analyses of fMRI data collected using the MSIT paradigm will focus on baseline activations in cingulo-opercular and frontoparietal (CC) as predictors of CBT response in the full sample, and test for associations between pre- to post-CBT changes in activation with changes in anxiety. Neural mechanisms of CBT effect will be tested with either ANOVA or multiple regression by examining treatment group (CBT, RMT) × time (pre-, post-treatment) interactions on change in anxiety severity. The 3-way interaction of treatment group × time × patient age will be examined to assess for developmental effects. We anticipate that greater activation in task control networks (i.e., greater CC capacity) at baseline will predict better CBT outcome, that greater increases in task control network activations following CBT (i.e., improved CC capacity) will relate to better CBT response and that these findings will be more pronounced in older patients, given prior work indicating a maturation of frontoparietal regions implicated in CC as children age [78,79]. Furthermore, to maximize our brain-behavioral data across more subjects during the MSIT, we have increased the number of runs from 3 to 5 for the additional subjects (approximately half the sample) to be collected in the next 1.5 years; this will allow us to preserve more subjects (at least five errors subject has to make in order to be included in the error-related analyses) when evaluating how pre- to post-CBT change in error-related brain activation may relate to treatment response.

Planned research will also include analysis of EFSAT data (AT, CC-AT). We hypothesize that lower AT at baseline (i.e., less reactive amygdala) will predict better CBT outcome, that greater reductions in AT following CBT will relate to better CBT response (i.e., reductions in amygdala reactivity from pre to post), and that these relationships will be more pronounced in younger patients, given work suggesting reduction in amygdala reactivity drives positive CBT response. Furthermore, greater CC-AT at baseline will predict better CBT outcome (i.e., greater PFC activation during “match shapes” condition of EFSAT), and greater increases in CC-AT following CBT will relate to better CBT response (i.e., *increase* in PFC activation and improved task accuracy during the “match shapes” condition); we would expect these relationships will be more pronounced in older patients, as greater CC capacity through activation of the PFC would result in better CBT response for these children, relative to younger children (who may demonstrate improvement as a function of reduction in amygdala reactivity).

Functional connectivity analyses will also be conducted to examine how brain connectivity underlying CC, AT, and CC-AT interaction relate to treatment response. Based on prior work, we would expect greater pre- to post-CBT decrease in anxiety severity is predicted by (1) more dACC-vLPFC connectivity during CC at baseline; (2) greater amygdala inverse connectivity with pgACC during CC-AT at baseline; (3) greater increase of dACC-vLPFC connectivity from pre- to post-CBT during CC; (4) greater increase of amygdala inverse connectivity with pgACC during CC-AT from pre- to post-CBT.

For the diffusion MRI, we will use probabilistic tractography to examine the degree of white matter connectivity between subcortical and specific PFC structures [80,81]. We hypothesize that greater white matter connectivity between the amygdala and PFC, including Brodmann’s Areas 47, 10 and 11, will predict better response to CBT and that CBT will relate to increased connectivity in these same tracts. Finally, we will use diffusion tensor imaging (DTI) to examine the uncinate fasciculus and other white matter bundles. We predict that fractional anisotropy, a DTI measure of fiber density, axonal diameter and myelination, will positively relate to CBT response.

To address the behavioral components of CC, AT, and CC-AT alongside our neuroimaging data, we will continue to draw upon performance measures (e.g., reaction time, error rates) on the MSIT and EFSAT. It will be critical to examine multi-level predictors of clinical outcomes separate from brain, and the interaction between brain and these behavioral variables, in order to full examine brain-behavioral constructs from within an RDoc framework [82,83]. Accordingly, we acknowledge that we cannot be certain that all brain-behavioral markers will cohere as a single construct (i.e., of AT, CC, or AT-CC) with collective power to predict CBT outcomes; thus, we will examine the standardized parameter estimates for each independent variable in each linear regression model to determine its relative contribution as a predictor of pre-to-post CBT change in PARS. If an independent variable(s) appears *not* to contribute to the model (based on *p*-value[s]), then we will use a stepwise model selection procedure to identify the strongest combination of predictors.

## STUDIES OF CLINICAL MECHANISMS OF CBT RESPONSE IN YOUTH ANXIETY

### Background & Methods

The current project also allows for examination of clinical and behavioral characteristics of a large sample of anxious youth, and how these characteristics associate with anxiety symptom severity and CBT outcomes. Such work can help identify children for whom CBT may be more or less effective.

In order to understand how CBT, an evidence-based treatment for anxiety and other disorders, affects subject clinical characteristics including anxiety severity, impairment, and improvement pre- to post-treatment, CBT must be compared to another treatment. The current study randomly assigned subjects in a 2:1 ratio to 12 weeks of CBT (specifically consisting of psychoeducation, cognitive restructuring, and exposure), or to a relaxation and mentorship training intervention (RMT). CBT protocols for anxious youth typically include psychoeducation, relaxation training, cognitive restructuring, and exposure therapy. Some research has indicated that relaxation training may not be as effective as either cognitive restructuring or exposure therapy for anxious youth, and may not be necessary in CBT [67,84]. Thus, the CBT treatment dropped relaxation from the protocol, and patients are moved more quickly to cognitive restructuring and exposure than in other models (e.g., Coping Cat). By contrast, the control treatment, RMT, incorporates relaxation training as well as other non-specific supportive elements, while excluding elements of exposure and cognitive restructuring. Clinicians (master’s level psychologists and social workers) received rigorous training and supervision in the models, and the first full courses of treatment (of both CBT and RMT) for each therapist were observed via audiotape by the clinical supervisor, when available. Therapist fidelity to treatment manuals is maintained in weekly meetings with a clinical psychologist (ELB) who is expert in the delivery of CBT for anxiety disorders and designed the therapy manuals for this study. Weekly supervision ensures we provide high quality care to participants across conditions. Subject adherence to both CBT and RMT treatments was measured by therapist ratings using a treatment adherence rating scale measuring the degree of completion and effort put forth on assigned therapy homework between sessions. Thus, the current study also provides opportunities to study active “ingredients” and of an exposure-focused CBT relative to a less active, relaxation-based control therapy (RMT). Further, inclusion of these two therapy conditions enables analyses testing whether clinical factors (e.g., patient engagement) differentially moderate or mediate clinical improvement for one therapy compared to the other.

### Progress Report

#### Study 1. Parent psychopathology and accommodation behaviors as predictors of child anxiety severity

Particular parental characteristics, including accommodation behaviors and symptoms of psychopathology, are thought to hinder youth success in treatment. Parental accommodation, or modifying behavior in order to reduce child distress, is frequently observed in parents of children with anxiety disorders [85]. Parent depressive and anxious psychopathology is also commonly associated with psychopathology in their children [86,87]. To date, there is sparse work exploring both accommodation and parent psychopathology in relation to symptom severity in clinically anxious children. In this study, parent psychopathology and accommodation behaviors served as predictors of childhood anxiety severity in 109 clinically anxious youth from the larger study. In a linear regression predicting anxiety severity from accommodation and parent anxiety and depressive symptoms, only accommodation emerged as a significant predictor, with greater parent accommodation predicting higher child anxiety severity. Preliminary results indicate that while parent psychopathology is related to child anxiety severity in correlational analyses, parents’ accommodation behaviors may be a more robust predictor of anxiety severity in our sample of clinically anxious youth, perhaps suggesting additional focus on these parenting behaviors may be useful in prevention and treatment contexts.

#### Study 2. Comparison of CBT and RMT treatment conditions on anxiety severity and improvement post-treatment

CBT for youth anxiety typically comprises of psychoeducation, relaxation, cognitive restructuring, and exposure therapy. However, evidence suggests that exposure and cognitive restructuring may be the most active ingredients in CBT [67,88]. Participants were 67 clinically anxious youth enrolled in the larger study (CBT; *n* = 47 or RMT; *n* = 20). Mixed-model repeated-measure ANOVAS were run to examine trajectory of treatment response over the course of 12 weeks of either CBT or RMT. A significant between-subject effects for Independent Evaluator’s report of child anxiety severity and clinical global severity emerged; post-hoc analyses revealed no pre-treatment differences between CBT and RMT on either anxiety severity or global symptom severity. At post-treatment, CBT was associated with significantly lower anxiety and global symptom severity, and CBT was associated with significantly higher global improvement ratings than RMT. Results indicate that psychoeducation, cognitive restructuring, and exposure were associated with significantly more improvement in symptoms of anxiety and overall global severity as compared to an intervention of relaxation and mentorship. This study provides some of the first evidence that CBT without relaxation can be more effective than relaxation strategies in relieving anxiety symptoms among clinically anxious youth.

#### Study 3. The differential impact of engagement on treatment outcome for youth with anxiety

Existing work has demonstrated that improvement following treatment with CBT is related to homework adherence [89]. Anxious youth (*n* = 51) from the sample were assigned homework across all 11 therapy sessions, and pertained specifically to exposure, cognitive restructuring for those enrolled in CBT (*n* = 37), and relaxation and non-exposure based activities for those enrolled in RMT (*n* = 17). Linear regression and correlational analyses indicated that for those enrolled in CBT, more adherence correlated with greater improvement in impairment scores post-treatment. However, for those in RMT, more adherence correlated with less improvement in impairment scores following treatment. Results suggest that homework engagement is important to CBT outcome, but perhaps only when homework consists of exposures. Future research should identify people most likely to struggle with homework adherence and consider methods for increasing engagement. The association of greater RMT adherence with worse outcomes raises the provocative question that greater practice of relaxation strategies, in the absence of cognitive restructuring or exposure, may hinder progress over 12 weeks.

### Presentations and Papers

- Hicks, A, Morrison, C, Lowe, R, Synger, A, Piacentini, J, et al. Exposure tasks and cognitive restructuring outperform relaxation training and mentorship in anxious youth. Poster presented at: the Association for Behavioral and Cognitive Therapies Annual Convention; 2018 Nov; Washington, DC, USA [90].
- Broner, S, Synger, A, Monk, CS, Phan, KL, Johnson, T, Peltier, S, et al. Engagement with therapy predicts treatment outcome among youth with anxiety. Poster presented at: the Fifth Annual Mental Health Research Symposium at the Michigan School of Psychology; 2019 Apr; Farmington Hills, MI, USA [91].
- Broner, S, Synger, A, Monk, CS, Phan, KL, Bilek, EL, Fitzgerald, KD. The differential impact of homework on treatment outcome for youth with anxiety. Poster presented at: the Association for Psychological Science Annual Convention; 2019 May; Washington, DC, USA [92].
- Bilek, EL, Broner, S, Lowe, R, Piacentini, J, Phan, L, Monk, CS, et al. Is relaxation necessary for improving anxiety symptoms among clinically anxious youth? To be presented at: the Anxiety and Depression Association of America Annual Meeting; 2020 Mar; San Antonio, TX, USA [93].
- Lowe, R, Premo, JE, Bilek, E, Monk, CS, Phan, KL, Fitzgerald, KD. Parent psychopathology and accommodation behaviors as predictors of child anxiety severity. Poster to be presented at: the Anxiety and Depression Association of America Annual Meeting; 2020 Mar; San Antonio, TX, USA [94].

### Planned Research

Recruitment for this study is ongoing, and additional work is underway to examine clinical and behavioral characteristics of the sample at baseline as predictors and covariates, as well as change across treatment, using ANOVA, as well as correlation and multiple linear regression techniques. Proposed analyses include a comparison of low-anxious and clinically-anxious youths on the performance of a response inhibition task (Go/No-Go); we hypothesize that clinically anxious youth would demonstrate significantly greater deficits in their ability to inhibit their response to incorrect stimuli than low anxious youth. Additionally, though DSM diagnoses are established for subjects for the clinically anxious-youth group via structured clinical interview, diagnosis is not explicitly a part of primary project aims but will be used in future studies to examining differences in subject response to treatment.

All preliminary work pertaining to differences between CBT and RMT, or components of treatment, will be examined within the full sample to maximize the impact of our work when published. To maximize the benefits of subject randomization, to avoid effects of non-random attrition, and to consider data from those who have received little to no treatment or varied in their adherence, we expect to conduct intention-to-treat analyses. In addition to preliminary work on patient treatment adherence described in Study 3 above, we may choose to examine implementation fidelity. To do so, an independent rater would evaluate a portion of clinicians’ session audiotapes. Future work with the expanded sample will also explore differences in therapist-, parent-, and child-reported response to treatment condition upon completion of the study. Further analyses will investigate whether cognitive restructuring and exposure alone have equivalent impact on symptoms as compared to benchmark studies of CBT for youth anxiety which include approximately 4 weeks of relaxation training (e.g., CAMS [95]).

## DISCUSSION

### Summary Statement and Current Results

We report on current findings and planned research from our grant-funded project, “Dimensional Brain Behavior Predictors of CBT Outcomes in Pediatric Anxiety”. We describe the larger goals and aims of the grant, and preliminary findings and planned work pertaining to (1) neural correlates of cognitive control, acute threat, and their interaction at baseline and following treatment, as well as (2) clinical and behavioral characteristics of our sample and effective components of treatment. Baseline fMRI findings for the Multisource Interference Task show greater activation of task control network regions in response to errors, including dACC and bilateral parietal cortex, associated with greater anxiety across a normal to abnormal range of severity. Among clinically anxious youth, greater error-related activation of the bilateral parietal cortex was found, providing new evidence of altered cognitive control function within the fronto-parietal network in the pediatric anxiety disorders. These findings are consistent with recent work in a large community sample of youth showing anxious-misery symptoms to associated with widespread hyperactivation of the executive network during a working memory task [50].

Although not originally designed to examine clinical predictors of anxiety severity and/or CBT effect, our study lends itself to answering important questions in these areas as well. Studies of clinical characteristics of our youth sample suggest parent accommodation exacerbates anxiety symptom severity. Further, our studies provide new evidence that CBT without relaxation can be more effective than relaxation strategies in relieving anxiety symptoms among clinically anxious youth. Interestingly, the practice of cognitive restructuring and exposure-based exercises improves youth outcomes, whereas the practice of relaxation *without* these other elements of CBT associated with worse outcomes. Future work will expand these findings and incorporate clinical characteristics into multi-modal brain-behavioral analyses to examine the most robust combination of predictors of CBT response.

### Importance of Planned Work

As predicted, interim neuroimaging analyses implicate baseline abnormality of task control network function with the expression of anxiety across the non-clinical to clinical range but, critically, pre- and post-treatment data are needed to assess the functional significance of these findings. For instance, should frontoparietal hyperactivation to errors resolve in clinically anxious youth who respond favorably to treatment, one might infer that baseline hyperactivity plays a pathological role in anxiety symptom expression and/or is driven by efforts to maintain task performance in the face of distraction by anxiety [52]; simultaneous consideration of both brain activation and on-task behavior (e.g., accuracy, response times) during fMRI scanning may help determine if this is the case. By contrast, should frontoparietal activation to errors further increase with CBT response, then we would infer that greater engagement of task control network may reflect a compensatory response by which anxious youth may overcome symptoms.

A key advantage of the ongoing study is the large targeted sample size (*N* = 280) and, while our primary analyses consider anxiety within a single dimension, we will also be able to explore possible effects of symptom subtypes. Recent work suggests biological distinctions between fear- and distress-related anxiety symptoms that can be measured using paradigms tapping acute threat and cognitive control constructs [96,97]. For example, recent work examined executive function (EF) in the Philadelphia Neurodevelopmental Cohort of 9498 youth, 8–21 years of age, and found an EF difference between the clinical domains of anxious misery and fear [50]. Specifically, lower EF across several EF components (attentional vigilance, response inhibition, conceptual flexibility, and working memory) associated with higher levels of anxious misery, whereas greater EF scores for attentional vigilance and working memory abilities were related to higher levels of fear. With 280 youth sampled to reflect the broad spectrum of anxiety, we will be able to explore whether particular “types” of anxiety differentially associate with brain-behavioral markers of cognitive control, acute threat and the implementation of cognitive control in the context of acute threat.

Importantly, by *combining* study brain-behavioral markers of CC, AT, and CC of AT, our study will help to inform RDoC hypotheses involving each of these constructs in the pathophysiology of anxiety. Moreover, by studying these RDoC constructs in the context of a clinical trial, we hope to move RDoC towards clinical translation. We do not expect that our final results will immediately lead to routine brain and/or behavioral assessments of clinic patients to guide treatment decisions, however, we do anticipate that our findings will pave the way towards the development of novel treatment strategies for patients who fail to respond to CBT.

Whether or not results from this study confirm our hypotheses or suggest alternatives, this research will guide future work to develop novel, brain-based strategies for the treatment of youth anxiety. For instance, should results show CBT response to be mediated by increases in task control network activation to cognitive control demands, then we would pursue the development of CBT augmentation strategies, such as cognitive training to augment CBT effect. Should reduction in amygdala reactivity to acute threat relate to CBT effect, then we would pursue novel strategies for desensitization training (or more rapid progression to exposure) to improve CBT outcomes. Should increases in prefrontal connectivity with amygdala at rest and/or during task mediate anxiety reduction after treatment, then we would test whether targeted application of attention-bias-modification training [98] increases frontolimbic connectivity for cognitive control in the context of acute threat and lower anxiety.

## ABBREVIATIONS

CBT: Cognitive Behavioral Therapy
fMRI: functional magnetic resonance imaging
DTI: diffusion tensor imaging
